# Building Overdose Data Linkage Capacity in US Public Health Departments: Challenges and Opportunities

**DOI:** 10.23889/ijpds.v9i5.2632

**Published:** 2024-09-18

**Authors:** Abigail Simms, Tera Beaulieu, Benny Michaud, Noel Tsui, Brandon Zagorski, Sarah Edwards

**Affiliations:** 1 ICES; 2 Métis Nation of Ontario; 3 University of Toronto

## Objective

Linking data on people who experience drug overdoses can provide new insights and opportunities for intervention. However, because public health data infrastructure in the United States (US) is often siloed, linkage activities can require incentivization and support. In 2023, we began funding 20 states to conduct overdose-related data linkage. We sought to understand baseline capacity in funded states.

## Approach

Data linkages in this project focus on two areas: 1) linking fatal and nonfatal overdose data and 2) linking fatal or nonfatal overdose data to prescription drug monitoring program (PDMP), criminal justice, or social determinants of health (SDOH) data. We report opportunities identified during implementation regarding states’ capacity to perform the required linkages as well as needs for additional training and technical assistance.

## Results

Of 20 funded states, 11 (55%) reported some previous experience linking fatal and nonfatal overdose data; 10 (50%) have linked PDMP data, while only 3 (15%) have linked SDOH data and 2 (10%) criminal justice data. Jurisdictional needs included: training on data linkage methods, including developing and validating linkage algorithms; overcoming administrative barriers to data sharing, such as data use agreements; and developing standardized approaches to characterizing events of interest in diverse data sources. States valued sharing strategies and experiences with each other.

## Conclusions/Implications

Additional capacity building is needed for states to successfully link and utilize overdose-related data. We are actively collaborating with state partners to facilitate peer-to-peer knowledge exchange to develop successful linkage programs.

